# The role of fatal family history and mode of inheritance in prostate cancer for long-term outcomes following radical prostatectomy

**DOI:** 10.1007/s00345-020-03147-6

**Published:** 2020-03-11

**Authors:** Valentin H. Meissner, Jamila G. H. Strüh, Martina Kron, Lea A. Liesenfeld, Stephanie Kranz, Jürgen E. Gschwend, Kathleen Herkommer

**Affiliations:** 1 Technical University of Munich, School of Medicine, Klinikum rechts der Isar, Department of Urology, Munich, Germany; 2 Institute of Epidemiology and Medical Biometrics, University of Ulm, Ulm, Germany

**Keywords:** Biochemical recurrence-free survival, Cancer-specific survival, Fatal family history, Mode of inheritance, Prostatic neoplasms, Radical prostatectomy

## Abstract

**Purpose:**

To determine whether fatal family history (FFH) or mode of inheritance in prostate cancer (PCa) has an impact on long-term outcomes following radical prostatectomy (RP).

**Methods:**

1076 PCa patients after RP with at least one deceased first-degree relative with PCa were included and stratified by FFH (four subgroups: fraternal, paternal, multiple, and none) and by mode of inheritance (two subgroups: male to male, non-male to male). We compared clinicopathological characteristics between subgroups with Fisher’s exact or Chi-square tests. Biochemical recurrence-free survival (BRFS) and cancer-specific survival (CSS) were analyzed using the method of Kaplan and Meier. Simple and multiple Cox regression with backward elimination were performed to select prognostic factors for BRFS and CSS.

**Results:**

Median age at surgery was 63.3 (range 35.9–79.4) years. The overall Kaplan–Meier estimated BRFS rate at 10 and 15 years was 65.6% and 57.0%, respectively. The overall Kaplan–Meier estimated CSS rate at 10 and 15 years was 98.1% and 95.7%, respectively. Neither FFH nor mode of inheritance were factors associated with worse BRFS. However, in multiple Cox regression, paternal FFH was an important prognostic factor for a better CSS (HR 0.19, CI 0.05–0.71, *p* = 0.014) compared to non-FFH.

**Conclusion:**

FFH and mode of inheritance do not seem to be prognostic factors of worse long-term outcomes following RP. Rather, a paternal FFH was associated with a better CSS; however, the reasons remain unclear. Nevertheless, patients after RP and FFH could be reassured that their own PCa diagnosis is not associated with a worse long-term outcome.

## Introduction

A positive family history (FH) of prostate cancer (PCa) is a well-known risk factor for PCa in addition to age and ethnicity [1–3]. Nevertheless, it remains difficult to inform PCa patients with a positive FH about the outcome of the disease. Although a positive FH has been found to be associated with earlier onset and lower grade tumors [4–6], there are conflicting results regarding long-term outcomes, i.e., biochemical recurrence-free survival (BRFS) and cancer-specific survival (CSS) [4, 7–11].

The strong genetic component of the disease additionally raises the question whether familial risks are higher for fatal than incident PCa. Investigating the impact of fatal family history (FFH) on the outcome after radical prostatectomy could provide a new approach towards better understanding the role of familial PCa and improve clinical counseling of affected patients. Indeed, previous studies from the Swedish cancer registry reported higher hazard ratios (HRs) of death from PCa in relatives of men who died from PCa [10, 11]. A recent American study reported on opposing outcome results: FFH was not associated with BRFS or clinicopathological characteristics compared to non-fatal or negative FH [12]. Since both studies had major limitations (lacked data on clinical characteristics and treatment [10] as well as insufficient sample sizes [10, 12] and follow-up periods [12]), it remains difficult to draw valid conclusions for patient counseling.

When investigating familial PCa, mode of inheritance is another important aspect that needs to be considered, as genetic susceptibility is a possible explanation for familial aggregation. To date, there are three known gene mutations (*BRCA 1*, *BRCA2*, and *HOXB13*) [13, 14] and several single-nucleotide polymorphisms [15] associated with an increased risk for PCa. However, this factor often remains neglected in the literature, although there is a need for further evaluation.

The objective of the present study was to analyze whether FFH or mode of inheritance is prognostic factors for long-term outcomes following radical prostatectomy, i.e., BRFS and CSS, and whether there are differences among the analyzed subgroups regarding clinicopathological characteristics.

## Materials and methods

### Database and study population

Data were obtained from the multicenter German Familial Prostate Cancer database consisting of more than 36,000 index patients and their relatives. Since 1993, this prospective study consecutively recruits and surveys newly diagnosed patients with PCa independent of the FH through cooperating clinics and urologists throughout Germany. Each year, patients provide information about sociodemographic and clinical characteristics as well as FH via questionnaires. Self-reported FH of PCa is verified by a histopathological report or a doctor’s letter. Verified, affected relatives are added to the database. Informed consent is obtained from each patient. More detailed descriptions of the database have been published previously [7, 16]

For the present analysis, we retrospectively identified 1248 patients with a first-degree FH and with at least one deceased first-degree relative affected with PCa to ensure a definite classification. Furthermore, patients with primary therapies of PCa other than radical prostatectomy (*n* = 153) or with neoadjuvant therapy (*n* = 19) were excluded and 1076 were left for further analysis.

Defined by the cause of death of the deceased first-degree relative with PCa, patients were subdivided into four FFH subgroups:

- (fraternal) fFFH: brother died of PCa
- (paternal) pFFH: father died of PCa
- (multiple) mFFH: at least two first-degree relatives died of PCa
- (none) nFFH: deceased first-degree relative(s) died of something other than PCa

Based on information on the patient’s pedigree, patients were subdivided into two mode of inheritance subgroups:

- male to male (MTM): paternal mode of inheritance (father affected)
- non-male to male (nMTM): maternal mode of inheritance (only brothers affected)

### Statistical analysis

All subgroups were compared with regard to the following clinicopathological characteristics using Chi-square tests or the Fisher’s exact test in case of low counts: Heritability of PCa according to the Johns Hopkins criteria [(1) PCa in at least three first-degree relatives, or (2) PCa in three consecutive generations, or (3) PCa in two first-degree relatives with an age of onset < 55 years] [17], age at surgery, PSA level at diagnosis, TNM classification, surgical margin, pathological Gleason score, adjuvant radiotherapy, and adjuvant hormone therapy. Pathological staging was classified or reclassified for patients diagnosed before 2002 using the UICC TNM classification 2002 for prostatectomy specimens.

Kaplan–Meier analysis was performed to determine the overall BRFS and CSS rates. Analyses were run overall and stratified by FFH and mode of inheritance. Survival rates at 5, 10, and 15 years were calculated with 95% confidence intervals (CI). Any of the potential prognostic factors (Table 1) for BRFS and CSS were examined using simple Cox regression. Multiple Cox regression with backward elimination (selection level 5%) was carried out to simultaneously assess potential prognostic factors. Hazard ratios (HR) with 95% confidence intervals and *p* values were calculated.

**Table 1:** Patient characteristics of the study population (*n* = 1076) and stratified by fatal family history of prostate cancer and mode of inheritance

Characteristics	Total	Stratified by FFH					Stratified by mode of inheritance		
		fFFH	pFFH	mFFH	nFFH	*p* value*	MTM	nMTM	*p* value*
Hereditary PCa						< 0.001			0.951
Yes, *n* (%)	412 (38.3)	58 (40.9)	109 (31.4)	39 (100.0)	208 (37.9)		322 (61.8)	90 (61.5)	
No, *n* (%)	664 (61.7)	84 (59.1)	238 (68.6)	0 (0.0)	340 (62.1)		520 (38.2)	144 (38.5)	
Age at surgery (years)						0.003			< 0.001
Median (range)	63.3 (35.9–79.4)	64.8 (50.5–79.4)	63.1 (35.9–77.6)	64.3 (50.1–73.9)	62.8 (40.6–77.3)		62.9 (35.9–77.6)	64.7 (47.8–79.4)	
≤ 55, *n* (%)	137 (12.7)	12 (8.5)	56 (16.2)	2 (5.1)	67 (12.2)		125 (14.9)	12 (5.1)	
> 55 to ≤ 65, *n* (%)	531 (49.4)	63 (44.4)	170 (45.0)	19 (48.7)	279 (50.9)		421 (50.0)	110 (47.0)	
> 65 to ≤ 75, *n* (%)	389 (36.1)	59 (41.5)	116 (33.4)	18 (46.2)	59 (35.8)		283 (33.6)	106 (45.3)	
> 75, *n* (%)	19 (1.8)	8 (5.6)	5 (1.4)	0 (0)	6 (1.1)		13 (1.5)	6 (2.6)	
PSA at diagnosis (ng/ml)						0.168			0.050
Median (range)	7.6 (0.8–222.5)	9.1 (1.71–50.0)	7.3 (1.2–107.0)	9.22 (1.2–65.6)	7.4 (0.8–222.5)		7.4 (0.9–222.5)	8.3 (0.8–50.0)	
≤ 4, *n* (%)	96 (9.9)	13 (10.3)	35 (11.0)	3 (9.1)	45 (9.2)		75 (9.8)	21 (10.3)	
> 4 to ≤ 10, *n* (%)	548 (56.6)	61 (48.0)	190 (59.7)	18 (54.5)	279 (56.8)		449 (58.6)	99 (48.8)	
> 10 to ≤ 20, *n* (%)	224 (23.1)	38 (29.9)	68 (21.4)	5 (15.2)	113 (23.0)		170 (22.2)	54 (26.6)	
> 20, *n* (%)	101 (10.4)	15 (11.8)	25 (7.9)	7 (21.2)	54 (11.0)		72 (9.4)	29 (14.3)	
Pathological tumor stage						0.279			0.528
pT2, *n* (%)	714 (67.5)	89 (63.6)	234 (68.8)	21 (55.3)	370 (68.5)		565 (68.4)	149 (64.2)	
pT3a, *n* (%)	206 (19.5)	28 (20.0)	57 (16.8)	10 (26.3)	111 (20.6)		154 (18.6)	52 (22.4)	
pT3b, *n* (%)	120 (11.3)	21 (15.0)	43 (12.6)	7 (18.4)	49 (9.1)		92 (11.1)	28 (12.1)	
pT4, *n* (%)	18 (1.7)	2 (1.4)	6 (1.8)	0 (0.0)	10 (1.8)		15 (1.9)	3 (1.3)	
Pathological node stage						0.475			0.270
Nx, *n* (%)	135 (12.6)	18 (12.7)	38 (11.0)	5 (12.8)	74 (13.5)		111 (13.2)	24 (10.3)	
pN0, *n* (%)	872 (81.0)	112 (78.9)	293 (84.4)	30 (76.9)	437 (79.7)		681 (80.9)	191 (81.6)	
pN1, *n* (%)	69 (6.4)	12 (8.4)	16 (4.6)	4 (10.3)	37 (6.8)		50 (5.9)	19 (8.1)	
Surgical margin									0.940
R0, *n* (%)	419 (79.7)	46 (80.7)	148 (81.8)	11 (78.6)	214 (78.1)	0.812	342 (79.7)	77 (79.4)	
R1, *n* (%)	107 (20.3)	11 (19.3)	33 (18.2)	3 (21.4)	60 (21.9)		87 (20.3)	20 (20.6)	
Pathological Gleason score									0.506
≤ 6, *n* (%)	447 (48.5)	58 (47.9)	149 (50.7)	9 (29.0)	231 (48.6)	0.001	354 (48.9)	93 (46.9)	
7 (3 + 4), *n* (%)	65 (7.1)	11 (9.1)	17 (5.8)	3 (9.7)	34 (7.1)		50 (9.9)	15 (7.6)	
7, *n* (%)	216 (23.4)	21 (17.4)	70 (23.8)	6 (19.4)	119 (25.0)		166 (22.9)	50 (25.2)	
7 (4 + 3), *n* (%)	89 (9.6)	15 (12.4)	25 (8.5)	11 (35.5)	38 (8.0)		66 (9.1)	23 (11.6)	
8–10, *n* (%)	105 (11.4)	16 (13.2)	33 (11.2)	2 (6.4)	54 (11.3)		88 (12.2)	17 (8.7)	
Adjuvant radiotherapy						0.459			0.741
Yes, *n* (%)	73 (8.8)	8 (5.6)	24 (6.9)	5 (12.8)	36 (6.6)		56 (6.6)	17 (7.3)	
No, *n* (%)	1003 (93.2)	134 (94.4)	323 (93.1)	34 (87.1)	512 (93.4)		786 (93.4)	217 (92.7)	
Adjuvant hormone therapy						0.001			0.005
Yes, *n* (%)	105 (9.8)	24 (16.9)	27 (7.8)	8 (20.5)	46 (8.4)		71 (8.4)	34 (14.5)	
No, *n* (%)	971 (90.2)	118 (83.1)	320 (92.2)	31 (79.5)	502 (91.6)		771 (91.6)	200 (85.5)	

*FFH* fatal family history, *f* fraternal, *p* paternal, *m* multiple, *n* none, *MTM* male to male, *nMTM* non-male to male, *PSA* prostate-specific antigen, *PCa* prostate cancer

^*^p value from Chi-square test/Fisher’s exact test

## Results

### Patient characteristics

1076 patients were included in the final analysis. Median age at surgery was 63.3 (range 35.9–79.4) years and median PSA at diagnosis was 7.6 (range 0.8–222.5) ng/ml. The median follow-up was 9.7 (range 0.3–26.3) years. More than a third of the patients (38.3%) met the Johns Hopkins criteria for hereditary PCa. Regarding mode of inheritance, 78.3% of the patients were categorized into the MTM group, and 21.7% into the nMTM group. Regarding FFH of PCa, 13.2% of the patients were categorized into the fFFH group, 32.2% into the pFFH group, 3.6% into the mFFH group, and 50.9% into the nFFH group (Table 1). The overall BRFS after 5, 10, and 15 years was 78.9%, 65.6%, and 57.0%, respectively. The overall 5-, 10-, and 15-year CSS was 99.3%, 98.1%, and 95.7%, respectively.

### Fatal family history

Men with an nFFH and a pFFH subgroups were younger at surgery (median: 62.8 and 63.1 years) compared to men with an fFFH and an mFFH (median: 64.8 and 64.3 years) (*p* < 0.001). Men with a pFFH were more often diagnosed with a lower pathological Gleason score (*p* = 0.001) and treated less often with adjuvant hormone therapy (*p* = 0.001) compared to the other subgroups (Table 1).

**Fig. 1:**
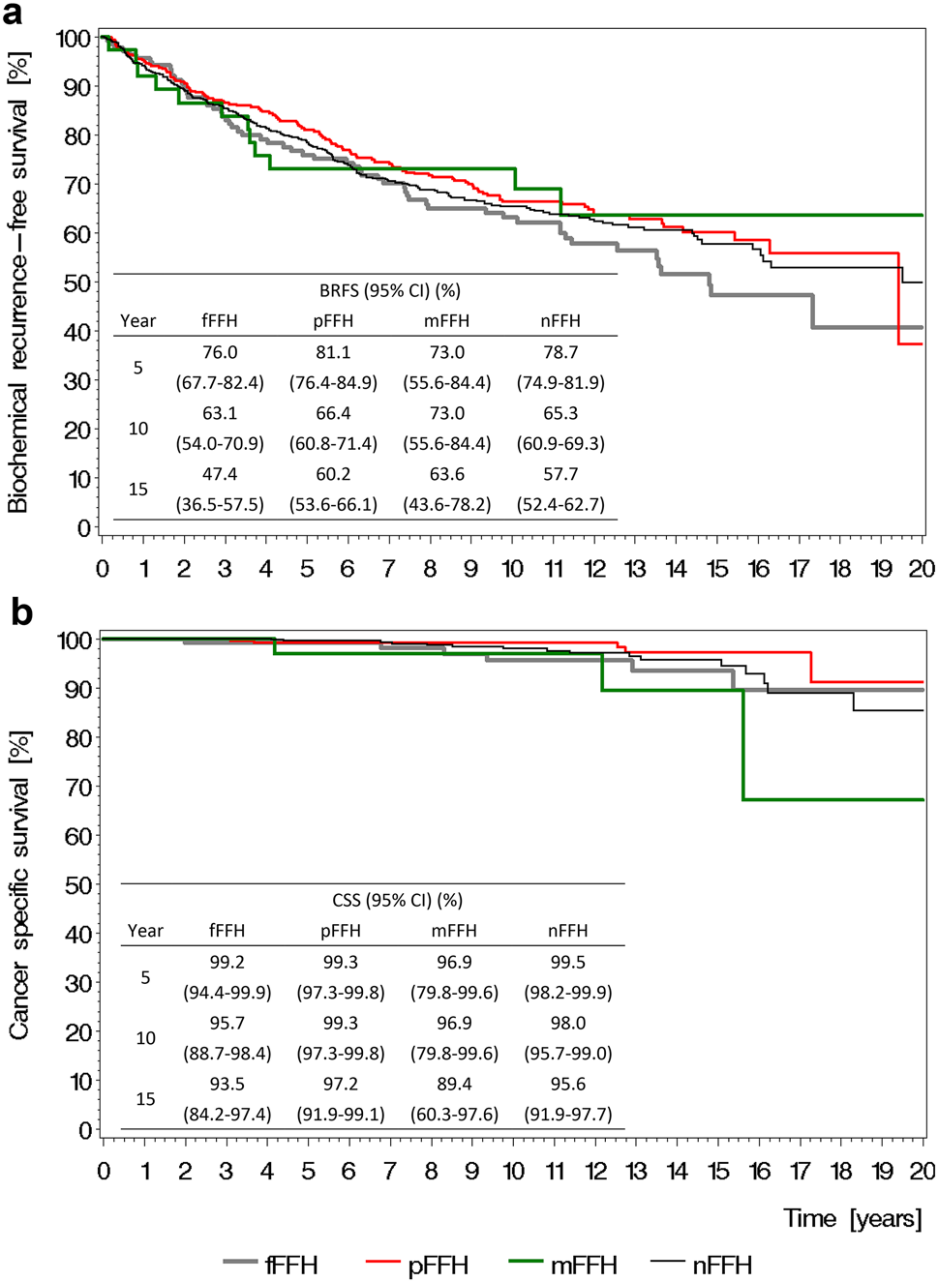
**a** Overall biochemical recurrence-free survival (BRFS) and **b** Overall cancer-specific survival (CSS) stratified by fatal family history of prostate cancer with 95% confidence interval (CI) (*FFH* fatal family history, *f* fraternal, *p* paternal, *m* multiple, *n* none)

Neither Kaplan–Meier estimated BRFS rates (Fig. 1a) nor CSS rates (Fig. 1b) differed among the four FFH subgroups.

In simple Cox regression, neither BRFS nor CSS were associated with FFH (Table 2). In multiple Cox regression, however, an important prognostic factor for CSS was FFH showing the lowest risk for pFFH (HR 0.19, CI 0.05–0.71) (Table 2).

**Table 2:** Simple and multiple Cox regression of potential prognostic factors for BRFS and CSS

Factors	BRFS						CSS					
	Simple Cox regression			Multiple Cox regression			Simple Cox regression			Multiple Cox regression		
	HR	95% CI	*p* value	HR	95% CI	*p* value	HR	95% CI	*p* value	HR	95% CI	*p* value
Hereditary PCa			0.023						0.131			
(Ref.: no)												
Yes	1.26	[1.03; 1.55]					1.71	[0.85; 3.42]				
Mode of inheritance			0.736						0.010			
(Ref.: MTM)												
nMTM	1.04	[0.82; 1.32]					2.50	[1.24; 5.04]				
Fatal family history of PCa			0.484						0.107			0.014
(Ref.: nFFH)												
fFFH	1.19	[0.89; 1.58]					1.58	[0.67; 3.71]		0.41	[0.12; 1.46]	
pFFH	0.93	[0.74; 1.16]					0.53	[0.19; 1.44]		0.19	[0.05; 0.71]	
mFFH	0.96	[0.55; 1.68]					2.62	[0.76; 9.01]		2.45	[0.68; 8.80]	
Age at surgery			0.073						0.115			
Continuous	1.02	[1.00; 1.03]					1.05	[0.99; 1.12]				
PSA at diagnosis (ng/mL)			< 0.001			< 0.001			0.111			
Continuous	1.02	[1.01; 1.02]		1.03	[1.02; 1.04]		1.02	[1.00; 1.04]				
Pathological tumor stage			< 0.001			0.028			0.001			0.011
(Ref.: pT2)												
≥ pT3a	2.44	[1.99; 2.98]		1.58	[1.05; 2.36]		3.36	[1.61; 6.99]		3.54	[1.34; 9.40]	
Pathological node stage			< 0.001			0.002			< 0.001			< 0.001
(Ref.: pN0/pNx)												
pN1	2.18	[1.58; 3.01]		3.36	[1.58; 7.15]		4.80	[2.15; 10.73]		7.27	[2.61; 20.27]	
Surgical margin			< 0.001			0.005			0.050			
(Ref.: R0)												
R1	2.12	[1.52; 2.95]		1.75	[1.19; 2.59]		4.95	[1.00; 24.57]				
Pathological Gleason score			< 0.001			< 0.001			< 0.001			
(Ref.: ≤ 6)												
7	2.02	[1.34; 3.06]		1.84	[0.98; 3.45]		1.16	[0.15; 9.28]				
7 (3 + 4)	1.67	[1.24; 2.25]		1.64	[1.04; 2.57]		2.37	[0.82; 6.86]				
7 (4 + 3)	2.69	[1.89; 3.82]		1.44	[0.71; 2.95]		7.09	[2.66; 18.89]				
8–10	4.09	[3.03; 5.53]		3.33	[2.05; 5.40]		5.64	[2.04; 15.64]				
Adjuvant radiotherapy			0.056						0.338			
(Ref.: no)												
Yes	1.41	[0.99; 2.01]					1.79	[0.54; 5.93]				
Adjuvant hormone therapy			< 0.001			0.035			0.006			
(Ref.: no)												
Yes	1.62	[1.22; 2.14]		0.47	[0.23; 0.95]		2.91	[1.35; 6.29]				

*BRFS* biochemical recurrence-free survival, *CSS* cancer-specific survival, *HR* hazard ratio, *CI* confidence interval, *MTM* male to male, *nMTM* non-male to male, *nFFH* non-fatal family history, *fFFH* fraternal fatal family history, *pFFH* paternal fatal family history, *mFFH* multiple fatal family history, *PSA* prostate-specific antigen

### Mode of inheritance

Men with a paternal mode of inheritance (MTM group) were younger at surgery (median: 62.9 vs. 64.7 years; *p* < 0.001) and had a lower PSA value at diagnosis (median: 7.4 vs. 8.3 ng/ml; *p* = 0.050) compared to men with a maternal mode of inheritance (nMTM group) (Table 1).

**Fig. 2:**
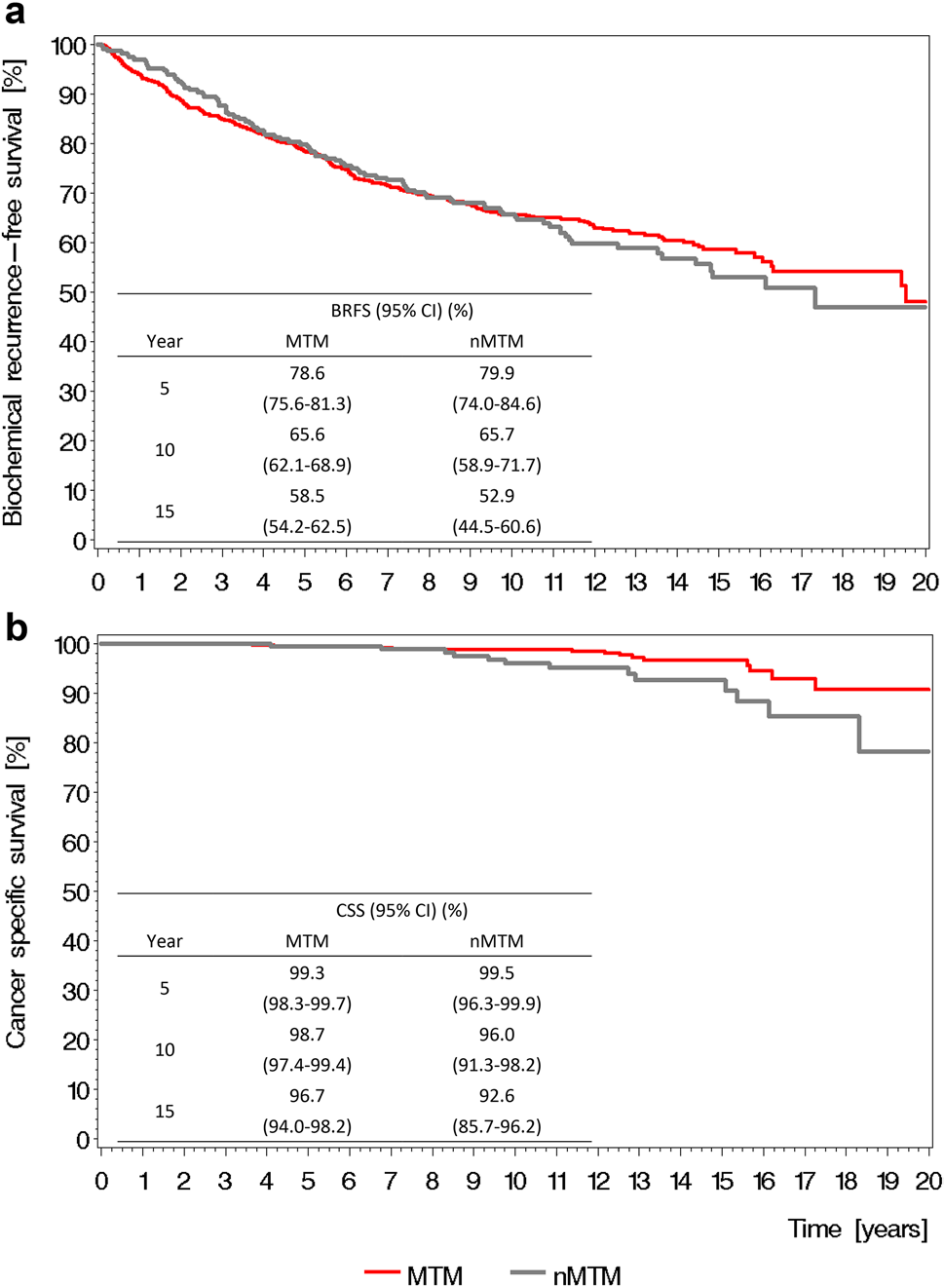
**a** Overall biochemical recurrence-free survival (BRFS) and **b** Overall cancer-specific survival (CSS) stratified by mode of inheritance with 95% confidence interval (CI) (*MTM* male to male, *nMTM* non-male to male)

The Kaplan–Meier estimated BRFS rate did not differ among the MTM and nMTM groups (Fig. 2a); however, the Kaplan–Meier estimated CSS rate was higher in the MTM group (Fig. 2b).

In the simple Cox regression, mode of inheritance was not associated with BRFS (*p* = 0.736), but nMTM mode of inheritance was associated with a worse CSS (HR 2.5, CI 1.24–5.04, *p* = 0.010) (Table 2). On multiple Cox regression, however, mode of inheritance was not selected (Table 2).

## Discussion

Aggressive PCa in a first-degree relative as well as an FFH of PCa might lead to anxiety in PCa patients. Thus, there is a need for profound medical counseling regarding long-term outcomes in these patients. As previous studies reported conflicting results whether an FFH of PCa increases the patient’s risk of dying due to PCa, the objective of this study was to analyze this factor in-depth in a large patient sample following radical prostatectomy [10, 12].

Our results are based on a large, representative German sample of 1,076 PCa patients after radical prostatectomy with a verified FH of PCa. The 5-year BRFS (78.9%) and CSS (99.3%) rates are comparable to those of another large, German collective of PCa patients after radical prostatectomy from a high-volume center (5-year BRFS: 76.9%; 5-year CSS: 99.0%) which confirms the representativeness [18].

In 2010, a study based on the Swedish cancer registry assessed for the first time whether the risk of dying from PCa is higher in men with an FFH. Brandt et al. reported higher HRs of death from PCa for men with a father or brother who died from PCa (HR 2.08, HR 2.30) compared to men with a negative FH of PCa. The highest HR was found when both father and brother died from PCa (HR: 6.86). However, HRs were assessed compared to men with a negative FH of PCa and not to an nFFH and the sample size was very low (pFFH: *n* = 202; fFFH: *n* = 15; men with a deceased father and brother *n* = 4) [10]. Hemminki et al. researched this topic 1 year later in 2011 and found that the incidence of PCa (HR 1.25) and the risk of dying from PCa (HR 1.28) increased for patients with a FFH of PCa compared to nFFH. Using nearly the same sample, the authors did not differentiate between different subtypes of FFH this time, and additionally, the lack of treatment and sociodemographic data make a valid comparison with our results hardly possible [11]. In contrast, an American study from 2014 found no association between an FFH and high-risk disease or biochemical recurrence in a collective of 471 men after radical prostatectomy. When interpreting these results, one should consider that the authors relied on insufficient sample sizes (patients with an FFH *n* = 19) and short follow-up periods (4–5 years).

Due to aforementioned findings, the role of FFH on long-term outcomes remains unclear. Therefore, we stratified FFH into four subtypes to evaluate this potential prognostic factor in detail. Moreover, the reference group consisted of patients with nFFH.

In multiple Cox regression, important risk factors for BRFS were especially pathological factors such as pathological tumor stage, pathological node stage, surgical margin, and pathological Gleason score. Neither FFH nor mode of inheritance was associated with BRFS.

Interestingly, our results showed that the Kaplan–Meier estimated CSS was slightly higher in patients with a pFFH compared to the other subgroups. Moreover, FFH was an important prognostic factor in the multiple Cox regression, with pFFH showing a better CSS compared to nFFH (HR: 0.19 CI 0.05–0.71). Unfortunately, using collected data, we cannot explain why a pFFH was associated with a better CSS. Earlier acquaintance of PCa due to a father’s PCa diagnosis and lethal outcome could lead to a healthier lifestyle or higher perceived risk and PCa worry, which are, indeed, associated with preventive health behaviors such as screening initiation [19].

We also investigated the role of mode of inheritance in PCa. Patients of the MTM group had better Kaplan–Meier estimated CSS as well as higher HR of CSS in the simple Cox regression. However, due to the fact that it was not selected in the multiple Cox regression, mode of inheritance is not an additional prognostic factor for long-term outcomes following radical prostatectomy. Hence, mode of inheritance (MTM vs. nMTM) might not be the ultimate proxy for gene mutations as their prevalence is very low [20, 21]. Therefore, we should keep in mind that the lack of association with long-term outcomes may not apply to patients predisposed to developing aggressive disease (e.g., BRCA carriers).

The strengths of our study are the large nationwide, population-based sample, detailed information about clinicopathological characteristics. Moreover, our data provide verified, complete, and in-depth data on FFH and mode of inheritance. On one hand, the fact that we only included patients following radical prostatectomy is a very rigorous patient selection, which allows excellent comparability and precise factor evaluation, but, on the other hand, this might cause a selection bias and does not allow us to make statements about patients with advanced tumor stages and inoperable settings. Furthermore, we neglected time between diagnosis and RP, since recently published studies did not show an impact on oncological outcomes [22, 23].

## Conclusions

Summarizing our result, we conclude that a positive FFH and mode of inheritance are not associated with worse long-term outcomes following radical prostatectomy. Conversely, a pFFH was rather an important prognostic factor for better CSS. Therefore, patients with deceased first-degree relatives due to PCa could be reassured that their own PCa diagnosis is not associated with a worse outcome if they are candidates for radical prostatectomy and undergo it.

## Abbreviations

BRCA 1,2: BReast CAncer 1,2
BRFS: Biochemical recurrence-free survival
CSS: Cancer-specific survival
FFH: Fatal family history
FH: Family history
f/p/m/n: Fraternal/paternal/multiple/none
HOXB13: Homeobox protein 13
MTM: Male to male
PCa: Prostate cancer
PSA: Prostate-specific antigen
RP: Radical prostatectomy
